# An integrated photonic circuit for color qubit preparation by third-order nonlinear interactions

**DOI:** 10.1038/s41598-022-09116-w

**Published:** 2022-03-25

**Authors:** A. L. Aguayo-Alvarado, F. Domínguez-Serna, W. De La Cruz, K. Garay-Palmett

**Affiliations:** 1 Departamento de Óptica - Centro de Investigación Científica y de Educación Superior de, Ensenada, BC 22860 México; 2 Cátedras Conacyt - Centro de Investigación Científica y de Educación Superior de, Ensenada, B.C. 22860 México; 3 Centro de Nanociencias y Nanotecnología, Universidad Nacional Autónoma de México, Km. 107 Carretera Tijuana-Ensenada, 22860 Ensenada, B.C. México

**Keywords:** Nanoscience and technology, Optics and photonics

## Abstract

This work presents a feasible design of an integrated photonic circuit performing as a device for single-qubit preparation and rotations through the third-order nonlinear process of difference frequency generation (DFG) and defined in the temporal mode basis. The first stage of our circuit includes the generation of heralded single photons by spontaneous four-wave mixing in a micro-ring cavity engineered for delivering a single-photon state in a unique temporal mode. The second stage comprises the implementation of DFG in a spiral waveguide with controlled dispersion properties for reaching color qubit preparation fidelity close to unity. We present the generalized rotation operator related to the DFG process, a methodology for the device design, and qubit preparation fidelity results as a function of user-accessible parameters.

## Introduction

Quantum information processing (QIP) involves manipulating and processing information coded on qubits. Several two-level systems have been proposed and implemented for qubit preparation, such as trapped ions^1^, quantum dots^2,3^, and superconducting materials^4^. However, single photons have been one of the most studied systems due to their more straightforward implementation, and relative lack of decoherence^5^. Broadly, these single-photon states are generated from parametric nonlinear processes, such as spontaneous parametric downconversion^6^ and spontaneous four-wave mixing^7^. Practical implementations of QIP tasks, like quantum computing, demand scalability of the quantum systems^8^, for which integrated micro and nanodevices become an attractive alternative^9–11^.

In generating single-photon-based qubits, the polarization basis has been the most exploited^12–15^; though, its two-dimensional Hilbert space does not allow harnessing the real potential of QIP. In recent years, different bases for qubit preparation have emerged as alternatives to the polarization basis. For example, the transverse spatial degree of freedom, through the orbital angular momentum (OAM) basis^16^, and the temporal/spectral degree of freedom in terms of the temporal mode (TM) basis^17^. Both schemes have attracted significant attention to encoding information since they reside in high-dimensional Hilbert spaces.

The temporal mode basis has advantages over the OAM basis since it can be used from pulsed optical fields in guided media and is less sensitive to disturbances^17,18^, establishing an excellent option for applications in QIP^19^. Recent work on quantum pulse gate^20–22^ has opened up a route for TM quantum logic implementation based on nonlinear interactions that involve frequency conversion of optical fields as an alternative to the extensively implemented quantum computing protocols by linear optics^23^. Temporal mode based qubits find applications, for instance, in quantum network nodes, where the frequency of flying qubits (photon states) can be converted (through a unitary transformation resulting from a nonlinear process) to a different frequency at which the stationary qubits (usually a material system) are sensitive^24^, but without altering the information coded in their quantum state^25,26^. The former kind of qubits is known as color qubits^27,28^, in the sense that the quantum state can be in one frequency or another, i.e., there is a two-level system that allows for the interaction of two frequencies $\omega _s$ and $\omega _r$, whose linear combination forms a qubit of the form $|\Psi \rangle = c_r|\omega _r\rangle + c_s|\omega _s\rangle$, where $|\omega _j\rangle$ is a single-photon state of frequency $\omega _j$ and $c_j$ its corresponding complex coefficient, with $j = {s,r}$.

**Figure 1:**
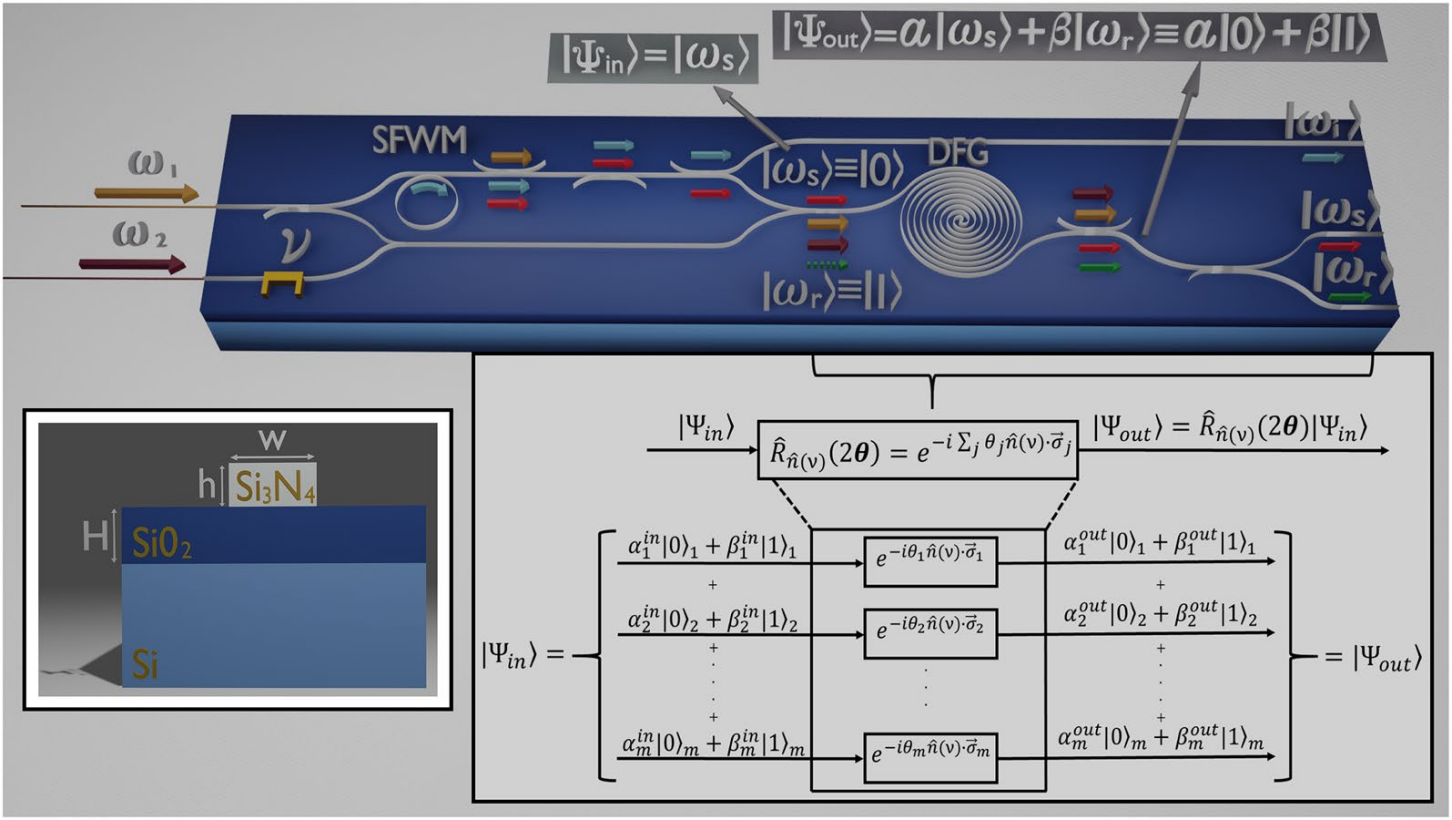
Design of the proposed integrated photonic circuit for color qubit preparation and rotations. All components in the device are based on silicon nitride (Si$_3$N$_4$) waveguides above a substrate of silicon dioxide (SiO$_2$) on silicon (Si). The top scheme describes the circuit operation, comprising the stage for generating a heralded single-photon state, which acts as input to the quantum gate waveguide (the second stage), where the DFG process occurs. The bottom-right scheme depicts the DFG process that leads to a generalized rotation operator for single-qubits defined in the temporal mode basis. Note that $|0\rangle = | \phi _j\rangle$ and $|1\rangle = | \psi _j\rangle$ are written on a computational basis in pairs of temporal modes. The bottom-left image is a representation of the waveguide’s geometry.

In this work, we propose a feasible integrated photonic circuit, capable of color qubit preparation (in the TM basis) and rotations by the third-order nonlinear process of difference frequency generation (DFG)^29^. Through this process, qubit rotations are restricted to axes lying in the *xy* plane of the Bloch sphere. The present work constitutes a step forward in the study that we recently published^28^. The proposed device (see Fig. 1) constitutes an example with high potential to be implemented, in which the demonstration of color qubit preparation by DFG with fidelities close to unity is achievable. The proposal comprises the on-chip integration of a heralded single-photon source by SFWM in a micro-ring cavity and a DFG source implemented in a spiral waveguide. Both sources have been engineered to increase the spectral overlap between the fundamental temporal modes of the input state (based on SFWM) and the DFG quantum gate, which lead to the desired qubit preparation fidelity. There are alternative mechanisms for controlling the spectral properties of the involved processes, such as those based on the group velocity matching technique^30,31^, through which similar results could be obtained and could also be implemented in integrated photonic circuits. In this sense, the presented theory and design methodology are general and can be applied in several configurations. Note that the TM basis for color qubit preparation demands unitary conversion efficiency and mode selectivity. However, in practical implementations reaching unitary efficiencies involve time-ordering corrections (ignored in the present treatment)^32,33^ that degrade the selectivity. This limitation can be overcome by implementing a cascading scheme based on an optical Ramsey interferometer^34,35^, a scenario we shall consider as future work.

## Results

This paper focuses on the third-order difference frequency generation process regarded as a unitary evolution for single-qubit preparation and rotations, which we propose implementing in the integrated photonic circuit of Fig. 1, designed under realistic conditions. We prove that the DFG process leads to a generalized rotation operator, around an axis with unit vector $\hat{\varvec{n}}(\nu )$, in the form $\mathbf{R} _{\hat{\varvec{n}}(\nu )}=\text{ exp }\left( -i\sum _{j}\theta _j\hat{\varvec{n}}(\nu )\cdot \hat{\varvec{\sigma }}_j \right) =\prod _jR^j_{\hat{\varvec{n}}(\nu )}(2\theta _j)$, where

1 $$\begin{aligned} R^j_{\hat{\varvec{n}}(\nu )}(2\theta _j)=\mathbb \mathbf{I }-\mathbb {I}_j+\text{ cos }\theta _j\mathbb {I}_j-i\text{ sin }\theta _j\left( \hat{\varvec{n}}(\nu )\cdot \hat{\varvec{\sigma }}_j \right) , \end{aligned}$$

with $\hat{\varvec{\sigma }}_j$ a vector of Pauli matrices, $\mathbb \mathbf{I }$ the identity matrix, $\mathbb {I}_j=|\phi \rangle \!_{j\,j}\!\langle \phi | + |\psi \rangle \!_{j\,j}\!\langle \psi |$, and $\hat{\varvec{n}}(\nu )=\langle \text{ cos }\,\nu ,\text{ sin }\,\nu ,0\rangle$. Each matrix $\hat{\sigma }^j_{x,y,z}$ couples the subspace *j*, $\{|\phi \rangle _j, |\psi \rangle _j\}$, such that each $R^j_{\hat{\varvec{n}}(\nu )}(2\theta _j)$ is valid for a pair of modes ($|\phi \rangle _j, |\psi \rangle _j$). For instance, $\hat{\sigma }_x$ in subspace *j* is written as $\hat{\sigma }_x^j = \vert \psi \rangle _j {}_j \langle \phi \vert + \vert \phi \rangle _j {}_j\langle \psi \vert$. Note $\mathbf{R} _{\hat{\varvec{n}}(\nu )}$ describes a rotation around an axis lying in the xy plane. The axis is defined by $\nu$, while the rotation angle $\theta _j$ varies for each *j*, which means that once the rotation axis is defined, all involved pairs ($|\phi \rangle _j, |\psi \rangle _j$) will rotate to a different angle. Therefore, after evolution, the output state will comprise in a linear combination several unwanted pairs, unless a single one interacts in a controlled manner^28^, a challenge we endeavored to overcome. Note that our treatment relies on the description of pulsed single-photon states in the temporal mode basis, such that $|\phi \rangle _j$ and $|\psi \rangle _j$ become to represent single-photon states in a single temporal mode. Also, note that in our proposal $\hat{\varvec{n}}(\nu )$ can be externally controlled, while $\theta _j$ will depend on the device geometry and experimental parameters.

Following the scheme in Fig. 1, the designed photonic circuit involves the generation of a heralded single-photon state by spontaneous four-wave mixing in a micro-ring resonator. The SFWM source is engineered for delivering a single-temporal-mode photon state $|\omega _s\rangle$, which becomes one of the two states of the computational basis (say $|0\rangle$). Then, this state is launched together with two pulsed pump fields (tuned at frequencies $\omega _1$ and $\omega _2$) into a waveguide, dispersion-controlled to fulfill phasematching for DFG at the desired frequencies. DFG can be defined as a frequency-translation process leading to the annihilation of $\omega _s-$photons and subsequent emission of single-photons at $\omega _r$, or the corresponding reversed interaction. The frequency conversion probability will depend on source parameters as the power of the pump fields. Note that in this description, a single-temporal-mode state $|\omega _r\rangle$ would correspond to the other state of the computational basis ($|1\rangle$).

The proposed DFG source is designed to favor the interaction in a particular sub-space *j*, i.e., to mediate the coupling between the temporal-mode pair ($|\phi \rangle _j, |\psi \rangle _j$). In such a case, even the DFG process involves the emission of a linear combination of temporal-modes pairs (as seen in Fig. 1), only the one matching the temporal mode of the single-photon input state will be efficiently delivered from the waveguide end. Roughly, the DFG nonlinear interaction can be described as a unitary evolution $\hat{U}$ that produces color qubits in the form $|\Psi \rangle _{out}=\hat{U}|\omega _s\rangle =\alpha |\omega _s\rangle +\beta |\omega _r\rangle$, with $|\beta |^2$ the probability of conversion and $|\alpha |^2=1-|\beta |^2$^28^. However, such description corresponds to the ideal situation for which the DFG unitary operator involves a unique temporal-mode pair ($|\phi \rangle _j, |\psi \rangle _j$), and also the input state. In general, both the input single-photon state and the DFG source comprise a linear superposition of temporal modes, which lead to unwanted interactions through the evolution in the nonlinear medium.

Our proposal is based on spectral-properties control engineering to let DFG interactions serve as a platform for qubit preparation and rotations. To quantify in a realistic scenario how close is the evolved state to the ideal one, we use a fidelity measure defined as $\mathcal {F} =\,^{\text {ideal}}_{\text {out}}\!\!\langle \Psi |\rho _{out}|\Psi \rangle _{\text {out}}^{\text {ideal}}$, where $\rho _{out}$ is the density operator denoting the evolved state.

Focusing on the ideal unitary transformation sketched in the top scheme of Fig. 1, the input state can be already in a superposition $\alpha |\omega _s\rangle +\beta |\omega _r\rangle$, previously prepared in an identical DFG source, which could be integrated to the same photonic circuit. On the other hand, let us highlight that once a color qubit is available, rotations, given in Eq. (1), are achievable for particular values of the phase difference ($\nu$) between the two pump fields. Thus, our proposal has the potential to implement, in an integrated monolithic device, the generation of a single-photon state, the preparation of qubits, and their rotations on the Bloch sphere.

### The proposed device

We propose an integrated photonic circuit for color-qubit quantum gate implementation based on silicon nitride rectangular waveguides lying above a plane substrate of silicon dioxide on silicon, as shown in the inset of Fig. 1^36^. The device integrates the stages for single-photon state generation by SFWM and the qubit preparation/rotation by DFG.

**Table 1:** Parameters of the SFWM and DFG sources of the proposed integrated photonic circuit

	SFWM	DFG
**Central wavelengths (μm)**		
Pump-1	$\lambda _{1}=0.822$	$\lambda _{1}=0.822$
Pump-2		$\lambda _{2}=1.554$
Signal-s	$\lambda _{s}=1.253$	$\lambda _{s}=1.253$
Signal-r		$\lambda _{r}=0.729$
Idler	$\lambda _{i}=0.612$	
**Waveguide dimensions (μm)**		
Core width	$w_{sfwm}=0.953$	$w_{dfg}=1.617$
Core height	$h=0.700$	$h=0.700$
SiO$_2$ height	$H=1.0$	$H=1.0$
Length	$l_c=43$	$L=1.0\times 10^4$
**Pump/filter bandwidths (THz)**		
Pump-1	$\sigma _1=6.0\,$	$\sigma _1=6.0\,$
Pump-2		$\sigma _2=0.7\,$
Filter	$\sigma _f=1.0\,$	
**Cavity reflectivity**		
	$$R_i=0.86$$	
**Nonlinear coefficient (mW)**^**−1**^		
	$\gamma _{fwm}=5.05$	$\gamma _{dfg}=2.50$

The single-photon state source consists of a micro-ring cavity resonant to the heralding mode (at frequency $\omega _i$). The design parameters for this source to herald a pure single-photon state (at frequency $\omega _s$) comprising just the fundamental temporal mode are summarized in Table 1. In turn, the DFG source is based on a waveguide engineered for integration with the single-photon source and reaching qubit-preparation fidelities close to the unit. The specific design parameters of the DFG source are also compiled in Table 1. Note that the two nonlinear interactions demand the use of waveguides with different widths ($w_{sfwm}$ and $w_{dfg}$), which waveguide tapers can interconnect with the minimum of losses by designing adiabatic transitions^37^.

The crucial point of our proposal is the attainment of qubit preparation fidelities close to unity. In this sense, the integration of the SFWM based single-photon source with the DFG source, as depicted in Fig. 1, becomes essential, which demands the simultaneous fulfillment of the phasematching constraint for the two nonlinear processes, at the same pump ($\omega _1$) and heralded single-photon ($\omega _s$) frequencies.

#### The heralded single-photon source

The SFWM-based source is proposed in a micro-ring cavity and is modeled according to the treatment given in reference^38^. In this configuration, a factorable two-photon state can be emitted if the cavity is resonant to at least one of the photons comprising the pair. In our case, we consider a cavity designed to be resonant to the idler photons, with wavelength $\lambda _i<\lambda _1$ ($\lambda _1$ being the the pump wavelength). The cavity-modified joint spectrum is composed of a set of cavity-allowed modes and, on the whole, exhibits spectral correlation, but the portion corresponding to each cavity-allowed mode is factorable. Then, by using a suitable spectral filter, we can choose a unique resonant mode (the central one in our case), obtaining a factorable two-photon state. The idler photons act as a trigger to herald the emission of signal photons, which are generally characterized by the density operator^39,40^

2 $$\begin{aligned} \begin{aligned} \rho _s&= \int d \omega _s \int d\omega _s' \int d \omega _i F(\omega _s, \omega _i) F^*(\omega _s',\omega _i) \hat{a}_s^\dagger (\omega _s) |\text {vac}\rangle \langle \text {vac}|\hat{a}_s (\omega _s'), \end{aligned} \end{aligned}$$

where $F(\omega _s, \omega _i)$ is the joint spectral amplitude (JSA) function of the cavity-based two-photon state and $\hat{a}(\omega ) \left( \hat{a}^\dagger (\omega )\right)$ is the annihilation (creation) operator of photons at frequency $\omega$. For accessing the temporal mode content of the two-photon state, the JSA is written in terms of the Schmidt decomposition^41^

3 $$\begin{aligned} \begin{aligned} F(\omega _s,\omega _i) = \sum _{m=1}^{\infty } \sqrt{D_m} \varphi _m (\omega _s) \Omega _m (\omega _i), \end{aligned} \end{aligned}$$

with $\varphi _m (\omega _s)$ and $\Omega _m (\omega _i)$ depicting two sets of temporal-mode basis functions, which are pairwise correlated^20^. The coefficients $D_m$ weighing the product of temporal-modes pairs are normalized such that $\sum _m\!\!D_m=1$. Note that an ideal factorable two-photon state involves only a temporal mode pair ($\varphi _m (\omega _s)$, $\Omega _m (\omega _i)$). In such a case, a detection event of trigger photons heralds the emission of a signal photon in a quantum-mechanically pure state; the purity defined as $p=\sum _m D_m^2$.

**Figure 2:**
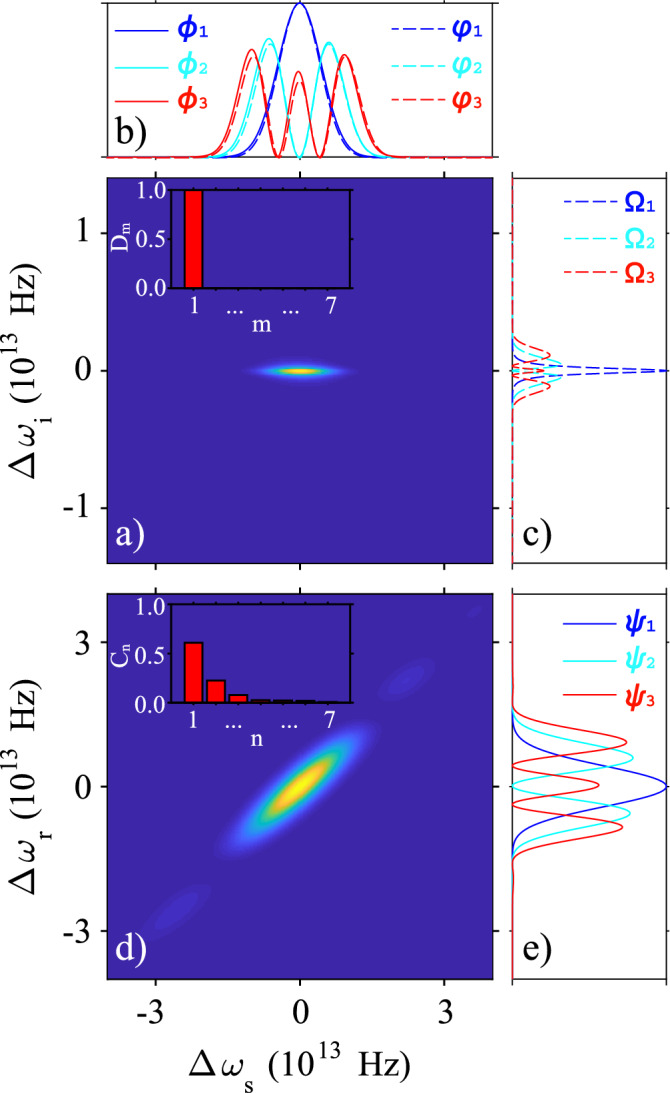
Spectral properties of the proposed SFWM and DFG sources. (**a**) The normalized joint spectral intensity of the SFWM source. (**b**) and (**c**) The three first temporal-mode pairs of the JSA ($\varphi _m (\omega _s)$, $\Omega _m (\omega _i)$), plotted as dashed lines. (**d**) The normalized DFG mapping function. (**b**) and (**e**) The three first temporal-mode pairs of the MF ($\phi _j (\omega _s)$, $\psi _j(\omega _i)$), plotted as solid lines. Note that the eigenfunctions have been normalized to the maximum value of the fundamental mode, while the joint spectral functions were normalized to their corresponding maximum value.The insets of panels (**a**) and (**d**) show the Schmidt coefficients of the JSA and MF, respectively.

Our proposal includes the implementation of an SFWM source in a micro-ring resonator of length $l_c$, which, after being pumped with pulses centered at $\lambda _1=2\pi c/\omega _1 = 0.822\,\mu$m is capable of delivering nearly uncorrelated photon pairs centered at wavelengths $\lambda _s=2\pi c/\omega _s = 1.253\,\mu$m and $\lambda _i=2\pi c/\omega _i = 0.612\,\mu$m. Then, detection of a visible photon (idler arm) announces an infrared photon in the signal arm, accessible for its use as input to the DFG source. The joint spectral intensity of the designed SFWM source, calculated as $|F(\omega _s, \omega _i)|^2$, is shown in Fig. 2a). Note that frequency axes are expressed as $\Delta \omega _{\mu }=\omega _{\mu }-\omega _{\mu 0}$, with $\mu =s,i$ and $\omega _{\mu 0}$ the central emission frequency. The joint spectrum exhibits the expected factorable feature, validated by calculating its Schmidt eigenfunctions and corresponding eigenvalues $D_m$. Figure 2b and c show the three first temporal-mode pairs ($\varphi _m (\omega _s)$, $\Omega _m (\omega _i)$), plotted as dashed lines, and the corresponding Schmidt eigenvalues are shown in the inset of Fig. 2a. As noticed, the JSA is essentially determined by the fundamental temporal-mode pair and consequently can be written as $F(\omega _s,\omega _i)\approx \varphi _1 (\omega _s) \Omega _1 (\omega _i)$. Hence, this SFWM source can lead to the preparation of a single-photon state with a spectral amplitude $\varphi _1 (\omega _s)$ and an estimated purity of 0.997. The reached level of photon-pair source factorability was obtained by adding a spectral filter modeled as a Gaussian shape bandpass filter of bandwidth $\sigma _f=1\,$THz and centered at the central idler emission frequency of the SFWM source. This filter enables the choice of a unique cavity-allowed mode (the central one in our case) among those comprising the cavity-modified joint spectrum^38^ while preserving a high photon-pair emission rate per pump-power squared, $\approx 0.4\,\text{ pairs }/\mu \text{ W}^2$. Integrated bandpass filters can be implemented from liquid crystals^42^ or microresonator systems^43^.

#### The DFG source for qubit preparation and rotations

The proposed DFG source is based on a $\chi ^{(3)}$ spiral rectangular waveguide, as shown in Fig. 1. In this process, a single-photon state at frequency $\omega _s$ ($\omega _r$) could be translated toward different frequency $\omega _r$($\omega _s$), through the interaction with two intense pump fields with frequencies $\omega _{1}$ and $\omega _2$ and phase difference $\nu$, constrained to energy and momentum conservation fulfillment^28^. This $\chi ^{(3)}$ nonlinear interaction is governed by an evolution operator given by

4 $$\begin{aligned} \hat{U} = \exp \left\{ \!- \zeta \sum _{k_s} \sum _{k_r} \left[ G(k_s,k_r) \hat{a}(k_s) \hat{a}^\dagger (k_r) + H.C. \right] \right\} , \end{aligned}$$

where $\zeta$ is a global constant, *H*.*C*. denotes hermitian conjugate, $k_s$($k_r$) is the propagation constant at $\omega _s$($\omega _r$), and $G(k_s,k_r)$ is the function that describes the spectral properties of the DFG process and is called onward mapping function (MF). An explicit form of this function, given in terms of frequencies rather than wavenumbers, can be found in reference^28^. Note that the MF squared is proportional to the probability of frequency conversion. Interestingly, the MF linearly couples the $\omega _s(k_s)$ and $\omega _r(k_r)$ modes during the interaction. The linearity of the DFG process has already been proposed as a method to obtain unitary transformations acting on qubits^18,21^, whose computational basis is formed of two single-photon states with different colors (frequency)^44^. This feature is the one we exploit for qubit preparation and rotations in our integrated photonic device.

As for the JSA (see Eq. 3), we write the MF in terms of the Schmidt decomposition $G(\omega _s,\omega _r) = \sum _{j=1}^{\infty } \sqrt{C_j} \phi _j (\omega _s) \psi _j (\omega _r)$, which leads us to rewrite the evolution operator of Eq. (4) as

5 $$\begin{aligned} \hat{U} = \text{ exp } \Big \{ -i \sum _j \Big [ \theta _j\hat{A}_j \hat{B}^\dagger _j+ \theta _j^*\hat{A}^\dagger _j \hat{B}_j \Big ] \Big \}, \end{aligned}$$

where time-ordering effects and other possible loss mechanisms were ignored; $\phi _j (\omega _s)$[$\psi _j (\omega _r)$] depicts the Schmidt eigenfunctions, and $C_j$ is the Schmidt eigenvalue. $\hat{A}_j$ and $\hat{B}_j$ represent temporal mode annihilation operators given by

6 $$\begin{aligned}&\hat{A}_j = \int d\omega _s \phi _j(\omega _s) \hat{a}(\omega _s), \end{aligned}$$

7 $$\begin{aligned}&\hat{B}_j = \int d\omega _r \psi _j(\omega _r) \hat{a}(\omega _r). \end{aligned}$$

The parameter $\theta _j$ in Eq. (5) couples the different pairs of temporal-mode eigenstates $|\phi (\omega _s)\rangle _j\equiv |\phi \rangle _j$ and $|\psi (\omega _r)\rangle _j\equiv |\psi \rangle _j$, and is given by

8 $$\begin{aligned} \theta _j = \epsilon \sqrt{C_j} L \gamma _{dfg} \sqrt{\frac{P_{av1}P_{av2}}{\sigma _1 \sigma _2}} e^{i\nu }, \end{aligned}$$

where $\epsilon$ is a global constant, *L* is the waveguide length, $\gamma _{dfg}$ is the nonlinear coefficient, and $P_{av\mu }$ and $\sigma _{\mu }$ are the average power and bandwidth of the pump field $\mu =1,2$, respectively. Beyond experimentally controllable variables, efficient coupling between a particular temporal mode pair is determined by the Schmidt coefficient $C_j$. Equations (5) to (8) reveal that the DFG process can enable the preparation of color qubits as linear combinations of the temporal-mode computational basis elements $|\phi \rangle _j$ and $|\psi \rangle _j$, in the form $|\Psi \rangle = \alpha _j |\phi \rangle _j + \beta _j |\psi \rangle _j$, see Fig. 1.

We conducted a parametrical numerical study that enabled us to identify the geometrical and pump characteristics for the DFG source, which are summarized in Table 1. The obtained spectral mapping function is shown in Fig. 2d, where frequency axes are also expressed in terms of detuning from the central frequencies. Figure 2b and e show the three first temporal-mode pairs ($\phi _j (\omega _s)$, $\psi _j (\omega _r)$), plotted as solid lines, and the first seven Schmidt eigenvalues are shown in the inset of panel 2d. In this case, the MF exhibits marked spectral correlation as several Schmidt pairs are needed to reconstruct the entire joint spectral function. The calculated Schmidt number characterizing this source is $(\sum _jC_j^2)^{-1}=2.33$. On the other hand, note in panel b) that functions $\varphi _1(\omega _s)$ and $\phi _1(\omega _s)$ spectrally overlap almost perfectly, which was the real challenge in the quest for the design of our integrated photonic circuit.

Let us now analyze the evolution of an input state through propagation along the DFG waveguide (quantum gate medium). Firstly, we assume a pure single-photon state as input, which can be expanded in the DFG temporal-mode basis $\{ \phi _j \}$ as $|\Psi \rangle _{in} = \sum _j \mathcal {O}_j \hat{A}^\dagger _j |\text {vac}\rangle$, where

9 $$\begin{aligned} \begin{aligned} \mathcal {O}_j=\int d\omega \phi _j^*(\omega )h(\omega ), \end{aligned} \end{aligned}$$

with $h(\omega )$ the spectral amplitude of the input state, normalized as $\int \! d\omega |h(\omega )|^2=1$, that can comprise several temporal modes. In the particular example of Fig. 1, the input state corresponds to the heralded single-photon at $\omega _s$ from the SFWM source, engineered for delivering a single-photon state in a single temporal mode, in which a case $h(\omega )$ would correspond to the first and prevailing eigenfunction in Eq. (3), i.e., $\varphi _1(\omega _s)$.

Through the nonlinear interaction with the two pulsed pumps, the input state $|\Psi \rangle _{in}$ will evolve to the output state^28^

10 $$\begin{aligned} \begin{aligned} |\Psi \rangle _{out}&= \hat{U}|\Psi \rangle _{in} =\mathcal {N} \Big ( |\Psi \rangle ^{\text {ideal}}_{\text {out}} + \sum _{j\ne 1} x_j \hat{A}_j^\dagger |\text {vac}\rangle + \sum _{j\ne 1} y_j \hat{B}_j^\dagger |\text {vac}\rangle \Big ), \end{aligned} \end{aligned}$$

where $\mathcal {N}$ is a normalization constant and $|\Psi \rangle ^{\text {ideal}}_{\text {out}} = \left( \cos \theta \,\hat{A}^\dagger -ie^{i\nu } \sin \theta \, \hat{B}^\dagger \right) |\text {vac}\rangle$, our interest’s color qubit, is the evolved state resulting from a perfect spectral overlap between a temporal-mode of the input state with one of the computational basis states $|\phi \rangle _j$ or $|\psi \rangle _j$, being the fundamental mode in our particular instance, see Fig. 2b. On the other hand, the terms $x_j= \mathcal {O}_j \cos \theta _j$ and $y_j= -i\mathcal {O}_je^{i\nu } \sin \theta _j$ arise from likely overlapping among the input state and DFG temporal-mode pairs beyond the fundamental pair. Accordingly, a linear combination of those evolved terms appears in the output state as a spurious contribution to the qubit.

Since our proposed input state is in a unique temporal mode and the DFG source has been spectrally engineered to guarantee an almost perfect overlap between its first temporal mode $|\phi (\omega _s)\rangle _1$ and that of $|\Psi \rangle _{in}$, the terms proportional to $x_j$ and $y_j$ in Eq. (10) are negligible, enabling to our interested qubit be quite distinguishable from the spurious background. The fidelity obtained under our source parameters is $\mathcal {F}=0.99$, a highly acceptable value for practical applications. Notice that despite the mapping function comprises several eigenfunctions, the fundamental pair makes the highest contribution, as can be seen in the inset of Fig. 2d. Our results prove that it is not required to factorize the mapping function nor use external control to favor the coupling of a unique temporal mode pair ($|\phi \rangle _j$, $|\psi \rangle _j$) and lead to an almost ideal color qubit preparation.

Let us remark that in a realistic situation, the heralded single-photon, acting as input to the quantum gate medium, will be in the mixed state $\rho _s$ given by Eq. (2), so that the density matrix describing the quantum gate can be calculated from Eq. (5) as $\rho _{out} = \hat{U} \rho _{s} \hat{U}^\dagger$, obtaining

11 $$\begin{aligned} \rho _{out}&= \hat{I} \cos ^2\theta |\omega _s\rangle \langle \omega _{s'}| + ie^{-i\nu }\hat{I}\cos \theta \sin \theta |\omega _s\rangle \langle \omega _r| - ie^{i\nu }\hat{I} \sin \theta \cos \theta |\omega _r\rangle \langle \omega _{s'}| +\hat{I}\sin ^2\theta |\omega _r\rangle \langle \omega _r|, \end{aligned}$$

where $\hat{I}$ is the double integral operator. Note this expression is less intuitive than the state in Eq. (10).

**Figure 3:**
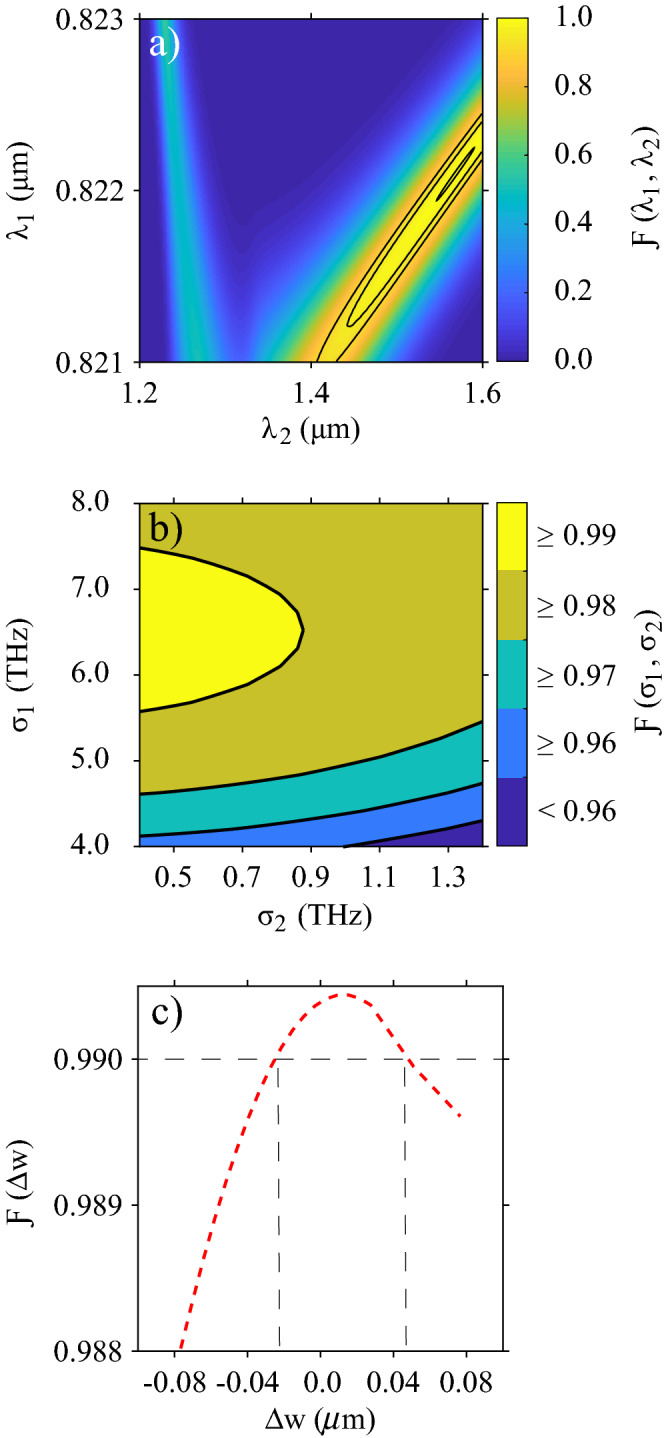
Color qubit preparation fidelity as a function of (**a**) the pump wavelengths, (**b**) the pump bandwidths, and (**c**) variations in the SFWM and DFG waveguide widths, while the remaining parameters of the SFWM and DFG sources are fixed to the values in Table 1.

## Discussion

As described in the previous section, our designed integrated photonic circuit allows the preparation of color qubit with high fidelity. A continuum of linear superpositions can be prepared by changing the coupling parameter $\theta _j$, which can be achieved by externally controlling the average power of the two pump fields. From Eq. (8), and under our proposed experimental conditions, we estimate that a complete translation of the single-photon state from $\omega _s$ to $\omega _r$ is achieved for a minimal product of the pump powers of 8.3 mW$^2$. A reasonable value to avoid distortion of signal spectra that can degrade our quantum gate’s fidelity, related to other nonlinear effects in the waveguides^45,46^.

Even though in the previously discussed example, we assumed an input state comprising components in just one dimension of the computational basis, in general, the input state to the DFG source may have the form $|\Psi \rangle _{in} = \sum _i \left( x_i \hat{A}^\dagger _i + y_i \hat{B}_i^\dagger \right) |\text {vac}\rangle$, for which the complete output state is calculated as $|\Psi \rangle _{out}=\mathbf{R} _{\hat{\varvec{n}}(\nu )}|\Psi \rangle _{in}$. Notice that the general input state can be prepared in an equivalent DFG source, as shown in Fig. 1, suggesting an additional but feasible stage in the same integrated photonic circuit. Likewise, as mentioned in the Results section, our proposed qubit logic is constrained to rotations around axes in the xy plane of the Bloch sphere, which can also be implemented in our designed photonic circuits by modulating the phase difference between the two pump pulses through an active integrated optical component^47^. However, note that entire Bloch sphere rotations could be realized by scaling our proposed device to allow consecutive rotations around the x and y axes, something entirely achievable by integrated photonic means, so its design is currently being explored.

Our proposed design, characterized by parameters given in Table 1, represents a feasible device to manufacture and characterize using the working group infrastructure. Still, in practical implementations, deviation from predicted conditions may occur, leading to undesired results. We estimate the quantum gate fidelity value as a function of pump wavelengths tunability and bandwidths and variations in the SFWM and DFG waveguide widths to validate our design’s robustness. Results are shown in Fig. 3. Surprisingly, from panel a) we can see a continuum of pump wavelength pairs for which fidelity reaches values close to unity. The three highlighted contours from external to internal correspond to fidelities higher than 0.9, 0.95 and 0.99, respectively. Note that for a wide $\lambda _2$ range (from 1.4 to 1.6 $\mu$m), the fidelity remains high enough for an appropriate $\lambda _1$ value tunable in a tight, narrow range. As noticed, the function $\mathcal {F}(\lambda _1,\lambda _2)$ exhibits two prominent stripes that tend to converge to a particular $\lambda _2$ value. In this convergency point, the DFG phasematching nontrivial solution and the two trivial phasematching solutions merge. For a fixed $\lambda _1$, the fidelity can reach higher values for longer $\lambda _2$’s (right stripe) than for $\lambda _2$’s shifted to the visible (left stripe). As explained in the previous section, the fidelity depends on the spectral overlap between the eigenfunction $\phi _1(\omega _s)$ and $\varphi _1(\omega _s)$ as dictated by $\mathcal {O}_j$ (see Eq. 9). Consequently, lower or negligible fidelity values (Fig. 3a) correspond to $\lambda _1$ and $\lambda _2$ pairs, leading to a diminished or null spectral overlapping. Note that trivial solutions represent the two cases i) $\omega _1=\omega _2$ and then $\omega _s =\omega _r$, and ii) $\omega _s=\omega _2$, and then $\omega _r=\omega _1$.

On the other hand, we evaluate the fidelity as a function of the bandwidths of the two pumps, $\mathcal {F}(\sigma _1,\sigma _2)$, while keeping the remaining parameters as described in Table 1. Results are displayed in Fig. 3b, where it is seen that fidelity values close to unity are obtained for narrower $\sigma _2$ values in the considered bandwidth range and $\sigma _1$ ranging approximately between 5.5 and 7.5 THz. Variations in the pump bandwidths affect the fidelity because the SFWM and DFG spectra change, leading to optimal or non-optimal spectral overlaps between the pair of eigenfunctions $\phi _1(\omega _s)$ and $\varphi _1(\omega _s)$, see Fig. 2 and Eq. (9).

Figure 3c shows the fidelity as a function of variations in the SFWM and DFG waveguide widths, rewritten as $w_{dfg} \rightarrow$ $w_{dfg} + \Delta w$ and $w_{sfwm} \rightarrow$ $w_{sfwm} + \Delta w$, with $\Delta w$ the deviation from the values reported in Table 1. We assume a simultaneous increase or decrease of the waveguide widths that can be originated, for instance, from a resolution variation in the system used to write the waveguides. The maximum width deviation considered in the simulation is $\Delta w = \pm 0.076 \, \mu$m. To calculate the fidelity, we fixed the other source parameters to the values reported in Table 1, except the operation wavelengths $\lambda _1$, $\lambda _s$, $\lambda _i$, $\lambda _r$, which must be adjusted for simultaneous perfect phasematching of the SFWM and DFG processes as waveguide dispersion properties modify with changes in the waveguide widths. It can be seen in the figure that fidelity remains close to unity for the explored $\Delta w$ range. Note there is an interval with a $0.07\,\mu$m width for which the fidelity is greater than 0.99, defined by the vertical dashed lines in Fig. 3c. The described analysis shows that our proposed design is robust against fabrication variations. Although the fidelity value varies if the waveguide widths result broader or thinner, the change is not so dramatic in an interval of more than 100 nm, while the operation wavelengths of the device remain experimentally accessible.

Results depicted in Fig. 3 reveal that once the proposed circuit has been implemented, there is flexibility in controlling its operation through external parameters related to the laser sources. They also indicate that if the geometrical parameters deviate slightly from the ideal values during the fabrication process, suitable quantum gate fidelities can still be obtained by choosing the proper phasematched wavelengths and pump bandwidth; a consequence of the SFWM and DFG spectral properties dependence on waveguide dispersion and pump characteristics.

**Figure 4:**
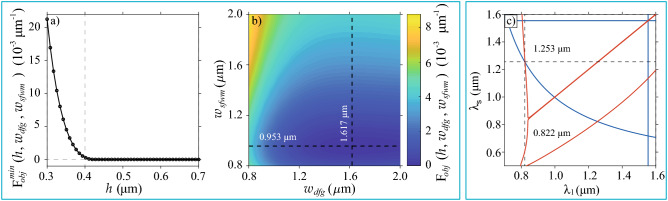
Method for simultaneous SFWM and DFG phasematching. (**a**) The minimum value of the objective function in terms of the core height *h*, when the widths of the two waveguides are allowed to vary. (**b**) Value of the objective function for $h = 0.7\,\mu$m in terms of the two waveguide widths. (**c**) Phasematching contours for SFWM (red) and DFG (blue), obtained with parameters $h = 0.7\, \mu$m, $w_ {sfwm} = 0.953\,\mu$m and $w_ { dfg} = 1.617 \,\mu$m.

In conclusion, we have designed a feasible integrated photonic circuit capable of color qubit preparation and rotations, based on the third-order nonlinear processes of spontaneous four-wave mixing and difference frequency generation. Our proposed device settles the basis for a photonic single-qubit logic relying on the temporal modes of pulsed single-photon states. It has been proved that by spectral engineering of the two nonlinear processes, assumed to be implemented in silicon nitride rectangular waveguides, qubit preparation fidelities close to unity are attainable. We consider our work a viable route for advancing quantum computing development by photonic means.

## Methods

For designing our integrated photonic circuit proposed to demonstrate color qubit preparation and rotations by DFG, we developed a parametric numerical exploration that enabled us to identify the set of physical parameters that lead to a quantum gate fidelity as close to unity as possible. For this, we propose an interaction in which the fundamental temporal modes of the input state and DFG quantum gate spectra almost perfectly overlap. This strategy requires satisfying the following conditions: (i) the JSA must be factorable, such that the heralded single-photon state contains only the fundamental mode, and (ii) although the mapping function can comprise several temporal modes, it must be such that only the fundamental one overlaps with that of the input state. Ideally, the overlap must be perfect for getting a unity fidelity, so the first step in our quest is to guarantee the simultaneous fulfillment of the phasematching conditions for the two processes (SFWM and DFG) at the same central frequencies $\omega _1$ and $\omega _s$, which depends on the dispersion properties of the nonlinear media.

**Figure 5:**
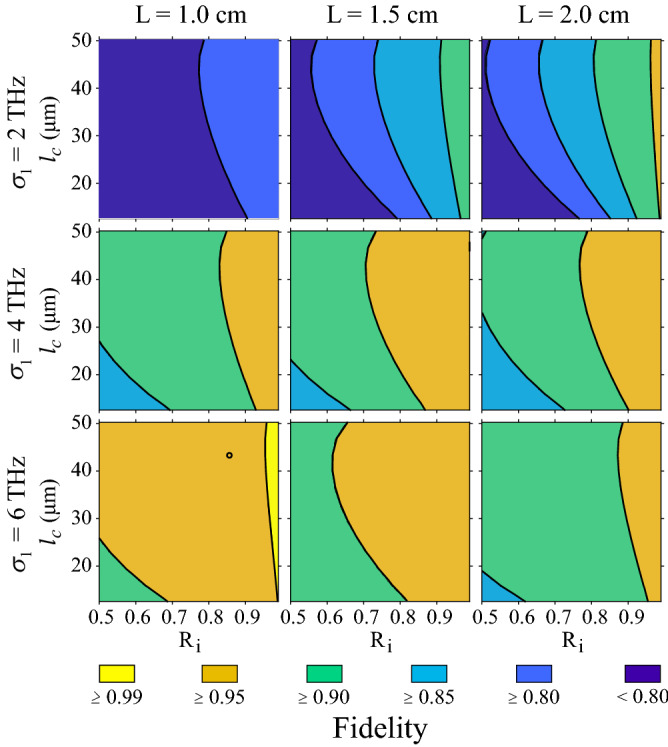
Methodology for increasing the qubit preparation fidelity. Single-qubit preparation fidelity in terms of the length of the DFG waveguide (*L*), the bandwidth of pump 1 ($\sigma _1$ ), and the length ($l_c$ ) and reflectivity ($R_i$) of the SFWM cavity, for $\sigma _2 = 0.5$ THz and $\sigma _f=4$ THz. The black circle marker indicates the combination of parameters we choose for our example.

Note that the material optical properties used in our numerical study correspond to those materials synthesized and characterized by the working group^36^, which become essential to make a real connection among simulations, experimental data, and feasible proposals. On the other hand, the waveguide dispersion properties were computed employing the opensource optical eigenmode solver WGMODES^48^, by using as free parameters the width (*w*) and the height (*h*) of the waveguide cores, while the height of the silicon dioxide layer was set to $1\, \mu$m.

**Figure 6:**
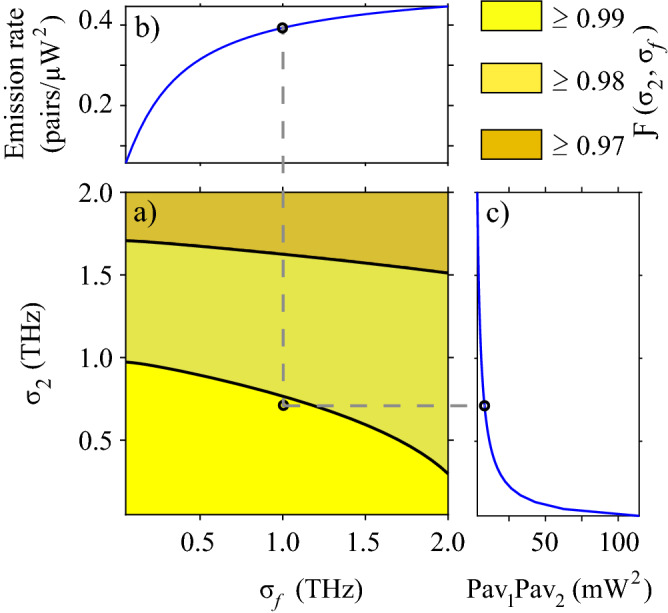
Methodology for increasing the qubit preparation fidelity. (**a**) Fidelity as a function of $\sigma _2$ and $\sigma _f$, while fixing the remaining parameters at the values corresponding to the black circle marker in Fig. 5. (**b**) The photon-pair emission rate per pump-power squared in terms of $\sigma _f$. (**c**) Minimum product of the average powers of the two pump fields required for a complete frequency conversion in terms of $\sigma _2$. The black circle markers correspond to the choice for our design.

To accomplish the conditions mentioned above, we proceed with the following methodology:

(i) To define the pump and signal wavelengths for the SFWM and DFG processes. In our case, taking into account our concern in an experimental demonstration of the proposed integrated circuit, we choose the central operation wavelengths of the device based on our availability of lasers sources and single-photon detectors for characterization.
(ii) To identify the waveguide geometrical parameters for the SFWM and DFG sources. For doing so, we define an objective function to be minimized, given by
  12 $$\begin{aligned} F_{obj}(h,w_{sfwm},w_{dfg})&= \Delta k_{sfwm}^2(h,w_{sfwm}) +\Delta k_{dfg}^2(h,w_{dfg}), \end{aligned}$$
  where $\Delta k_{sfwm}(h,w_{sfwm})$ $[\Delta k_{dfg}(h,w_{dfg})]$ represents the phase mismatching for SFWM [DFG]. Perfect phasematching requires the conditions $\Delta k_ {sfwm} (h, w_ {sfwm}) = 0$ and $\Delta k_ {dfg} (h, w_ {dfg}) = 0$ to be fulfilled, which reduces our problem to find the values of *h*, $w_ {sfwm}$ and $w_ {dfg}$ that minimize the objective function at the wavelengths of interest. We consider that fields in each waveguide propagate in the fundamental electrical transverse mode. The exploration ranges for each dimension were limited to realistic and feasible manufacturing values under our experimental conditions. Figure 4a shows the minimum value of the objective function in terms of the core height *h*, while the widths of the two waveguides are allowed to vary. It is interesting to see that for a certain value of the core height, $h\approx 0.4\,\mu$m, the function $F_ {obj} (h, w_ {sfwm}, w_ { dfg})$ reaches a plateau near the desired value $F_ {obj} (h, w_ {sfwm}, w_ {dfg}) = 0$. The observed behavior suggests that for each *h* on that plateau, there is a combination of $w_ {sfwm}$ and $w_ {dfg}$ values, for which phasematching is simultaneously achieved for the two processes. Figure 4b shows the value of the objective function for $h = 0.7\,\mu$m in terms of the waveguide widths. It is worth noting that the minimum value shifts to lower $w_ {dfg}$ as the height decreases, while $w_ {sfwm}$ remains almost constant. Figure 4c shows the phasematching diagrams for the two processes, obtained with parameters $h = 0.7\, \mu$m, $w_ {sfwm} = 0.953\,\mu$m and $w_ { dfg} = 1.617 \,\mu$m. In the case of DFG the diagram is generated for $\lambda _ {2} = 1.554\,\mu$m. This figure shows that phasematching is fulfilled simultaneously for the two processes at $\lambda _1=0.822 \,\mu$m and $\lambda _s=1.253 \,\mu$m.
(iii) To boost the quantum gate fidelity through variations of user-accessible parameters. By keeping fixed the bandwidths of pump 2 ($\sigma _2 = 0.5$ THz) and the spectral filter in the heralding arm of the SFWM source ($\sigma _f=4$ THz), we evaluate the fidelity as a function of the remaining device parameters in order to identify conditions for boosting the fidelity value. The free variables were the length of the DFG waveguide (*L*), the bandwidth of pump 1 ($\sigma _1$), and the length ($l_c$) and reflectivity ($R_i$) of the SFWM cavity, according to the model in reference^38^. The parameters were varied in ranges of values accessible experimentally. Results are displayed in Fig. 5 as a $3\times 3$ matrix, where columns correspond to three different values of *L*, while rows correspond to three different values of $\sigma _1$. Each matrix element shows the fidelity value as a function of $l_c$ and $R_i$. Results are presented as filled contours, with six levels indicating different fidelities ranges; see the colorbar underneath the matrix. As the figure shows, in our conditions, the fidelity increases for shorter *L*, wider $\sigma _1$, longer $l_c$, and high-quality cavities (as dictated by the $R_i$ value); those parameter combinations favor the spectral overlap between the fundamental temporal modes of the input state and the mapping function characterizing the quantum gate medium. We choose the parameters corresponding to the black circle marker on the bottom-left matrix element for the example discussed here and presented as a potential proposal to be implemented, i.e., $L=1$ cm, $\sigma _1=6$ THz, $l_c=43\,\mu$m and $R_i =0.86$, which lead to a fidelity $\mathcal {F}=0.983$. As the last step in defining our design, we vary the bandwidths $\sigma _2$ and $\sigma _f$ while fixing the remaining parameters at the values corresponding to the black circle marker in Fig. 5. Results are shown in Fig. 6. In addition to fidelity (panel a), we present results of the SFWM photon-pair emission rate (panel b)) and the minimum product of the average powers of the two pump fields (panel c) required for a complete evolution from the mode $\hat{A}^\dagger$ to the mode $\hat{B}^\dagger$, see Eqs. (6) and (7), since they are affected by $\sigma _2$ and $\sigma _f$. A high photon-pair emission rate is beneficial for getting reasonable heralding efficiencies of the SFWM based single-photon source, and a low minimum value of the product $P_{av1}P_{av2}$ is required for avoiding undesirable nonlinear effects in the DFG waveguide. Note that in the exploration ranges, the obtained fidelity values exceed 0.97 and can achieve values close to unity for smaller $\sigma _2$ and $\sigma _f$ at the expense of a reduced photon-pair emission rate and higher values of the product of the pump powers. For our proposed design, we set $\sigma _2=0.7$ THz and $\sigma _f = 1$ THz, which lead to a fidelity $\mathcal {F}=0.99$, a photon-pair emission rate per pump-power squared of $\approx 0.4$ pairs/$\mu$W$^2$ and a minimum product of the pump powers of 8.3 mW$^2$. In Fig. 6, the black circle marker indicates this particular result.

The above-described methodology allowed us to find a feasible design with high potential to be developed with currently available technology for photonic integrated circuits. The full description of our proposed circuit for qubit preparation and rotations is summarized in Table 1.

## Data Availability

Data underlying the results may be obtained from the authors upon reasonable request.
